# Generation of clade- and symbiont-specific antibodies to characterize marker molecules during Cnidaria-*Symbiodinium* endosymbiosis

**DOI:** 10.1038/s41598-017-05945-2

**Published:** 2017-07-14

**Authors:** Kao-Jean Huang, Zi-Yu Huang, Ching-Yen Lin, Li-Hsueh Wang, Pin-Hsiang Chou, Chii-Shiarng Chen, Hsing-Hui Li

**Affiliations:** 1 Institute of Biologics, Development Center for Biotechnology, New Taipei City, 22180 Taiwan; 2 Graduate Institute of Marine Biology, National Dong-Hwa University, Pingtung, 94450 Taiwan; 3 Department of Life Science, National Dong Hwa University, Hualien, 97401 Taiwan; 4 Taiwan Coral Research Center, National Museum of Marine Biology and Aquarium, Pingtung, 94450 Taiwan; 5 Department of Marine Biotechnology and Resources, National Sun Yat-Sen University, Kaohsiung, 80424 Taiwan

## Abstract

The endosymbiosis between cnidarians and dinoflagellates is responsible for the formation of coral reefs. Changes in molecules have been identified during the process of cnidaria-*Symbiodinium* endosymbiosis. However, the complexity of the molecular interaction has prevented the establishment of a mechanistic explanation of cellular regulation in this mutualistic symbiosis. To date, no marker molecules have been identified to specifically represent the symbiotic status. Because the endosymbiotic association occurs in the symbiotic gastrodermal cells (SGCs), whole cells of isolated SGCs were used as an antigen to generate monoclonal antibodies (mAb) to screen possible molecular candidates of symbiotic markers. The results showed that one of the generated monoclonal antibodies, 2–6F, specifically recognized clade C symbiotic *Symbiodinium* but not its free-living counterpart or other *Symbiodinium* clades. The expression levels of 2–6F mAb-recognized proteins are highly correlated with the symbiotic status, and these proteins were characterized as *N*-linked glycoproteins via treatment with peptide N-glycosidase F. Furthermore, their glycan moieties were markedly different from those of free-living *Symbiodinium*, potentially suggesting host regulation of post-translational modification. Consequently, the 2–6F mAb can be used to detect the symbiotic state of corals and investigate the complex molecular interactions in cnidaria-*Symbiodinium* endosymbiosis.

## Introduction

Coral reefs are the most diverse and productive marine ecosystems, and their trophic and structural foundation relies on cnidarian-dinoflagellate endosymbiosis^1^. Endosymbiotic interactions are widespread in marine environments, and one of the most noticeable symbioses is that between cnidarian hosts (corals and sea anemones) and dinoflagellates (e.g., *Symbiodinium* spp.). During the symbiotic state, *Symbiodinium* provides its photosynthetic products (glycerol, glucose, amino acids, esters, alcohols, and lipids) to the cnidarian^2^. Recently, coral reefs have experienced global ecosystem decline due to global warming, ocean acidification, and overexploitation^3^. These drastic environmental changes have led to the breakdown of the symbiotic relationship. Therefore, it is a priority to identify molecular markers that could be used specifically to monitor coral health.

Recently, genes associated with symbiosis have been explored using new genomic and transcriptomic technologies^4–12^. These symbiosis-associated genes are involved in various functions, such as pattern recognition, cell adhesion, vesicular trafficking, apoptosis, nutrient and metabolite transport, lipid storage and transport, and reactive oxygen species regulation^4^. However, fewer symbiosis-associated proteins have been identified through proteomic analyses^13–15^. Although these proteins have been identified as having different profiles in symbiotic and aposymbiotic states, they were found to be expressed in both states, not only in the symbiotic state. Symbiotic markers are defined as specific molecules (such as RNA, proteins, lipids and glycans) that are present only in the symbiotic state and not in the non-symbiotic state. They may be distributed in hosts, symbionts, or both. The identification of these markers may provide both the tools and insight necessary to elucidate the regulatory mechanism of endosymbiosis.

Symbiotic gastrodermal cells (SGCs) are unique host cells harboring the symbiotic *Symbiodinium* (i.e., the symbiont). During the initial step of the endosymbiotic process, SGCs are involved in the recognition and phagocytosis of *Symbiodinium* ^16, 17^. Proteomic analyses have demonstrated that SGC plasma membrane proteins are involved in the molecular chaperone/stress response, cytoskeletal remodeling, and energy metabolism^18^. Accordingly, SGCs have high potential to express symbiotic markers. Many types of whole cells have been used as antigens to immunize mice and generate antibodies that could recognize cell-surface antigens, such as human astrocytomas^19^, human B lymphoblastic leukemia cells^20^, Burkitt’s lymphoma tumor cells^21^, sheep red blood cells^22^, and killed whole cells of *Streptococcus mutans* ^23^. Therefore, in the present study, whole SGCs were used as the antigen to generate symbiosis-specific antibodies for possible symbiotic markers to investigate the characteristics of cnidarian-dinoflagellate endosymbiosis.

## Results and Discussion

### The generation and screening of monoclonal antibodies

**Figure 1:**
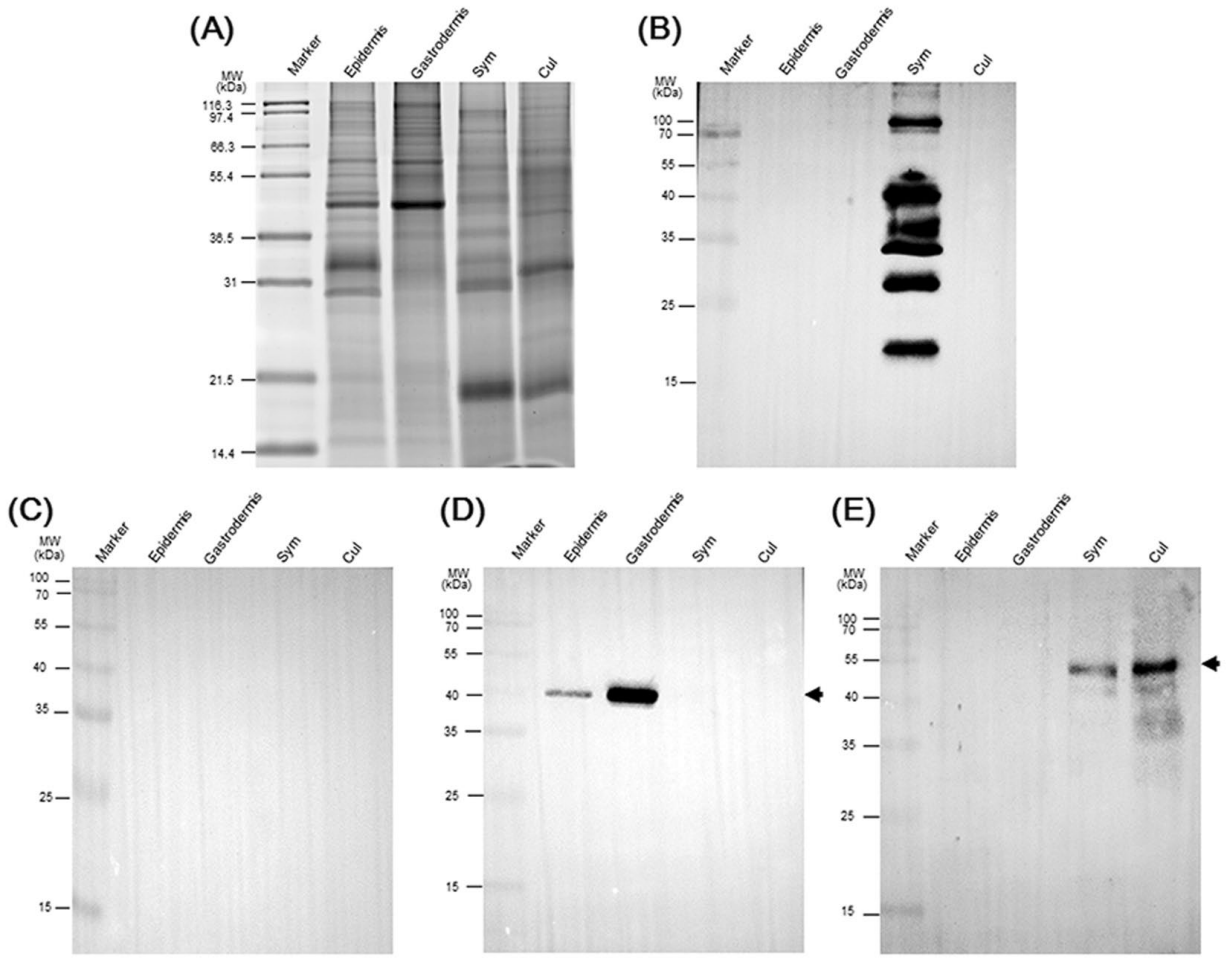
The 2–6F mAb only recognized symbiotic *Symbiodinium* proteins and not free-living *Symbiodinium* proteins. (**A**) Four types of protein were analyzed by SDS-PAGE, and total proteins were stained with SYPRO^®^ Ruby. Western blotting was performed using (**B**) the 2–6F mAb, (**C**) pre-immune serum, (**D**) anti-actin antibodies, and (**E**) anti-rubisco antibodies. In (**D**), the arrow indicates the actin protein (only present in host cells) with a molecular weight of 43 kDa. In (**E**), the arrow indicates the rubisco protein (only present in plant cells) with a molecular weight of 52 kDa. Epidermis: epidermis proteins from *E*. *glabrescens*; Gastrodermis: gastrodermis proteins from *E*. *glabrescens*; Sym: total proteins from freshly isolated *Symbiodinium* from *E*. *glabrescens*; Cul: total proteins of free-living clade C *Symbiodinium* (CCMP2466).

After screening monoclonal antibodies, the ELISA results showed 56 positive clones producing antibodies against total SGCs (data not shown). We then performed western blotting to examine the tissue targets of the antibodies produced by these clones. The purities of different tissues were assessed using an anti-actin antibody (coral host cell-specific) and anti-rubisco antibody (symbiont-specific) as shown in Fig. 1D,E (see also refs 24 and 25). Proteins from different cell lysates, including the whole epidermis, host gastrodermis, isolated symbionts, and cultured free-living *Symbiodinium* (CCMP2466, also clade C, the same clade as *Symbiodinium* of *E*. *glabrescens*), were then analyzed. Among these 56 clones, one clone, 2–6F, was chosen for further investigation due to its specificity, as shown in Fig. 1B. We did not obtain any clones that specifically recognized the gastrodermis. The 2–6F mAb exhibited high selectivity and specificity and only recognized symbiotic *Symbiodinium* (i.e., the symbiont) proteins and not cultured *Symbiodinium*, host cells, or epidermis (Fig. 1B). To examine the specificity of the 2–6F mAb, we used pre-immune serum (serum collected from mice before boosting with antigen) to perform western blotting. No positive signals were detected (see Fig. 1C), suggesting that the 2–6F mAb is specific.

### Characterization of the 2–6F mAb

#### Cell and tissue specificities

**Figure 2:**
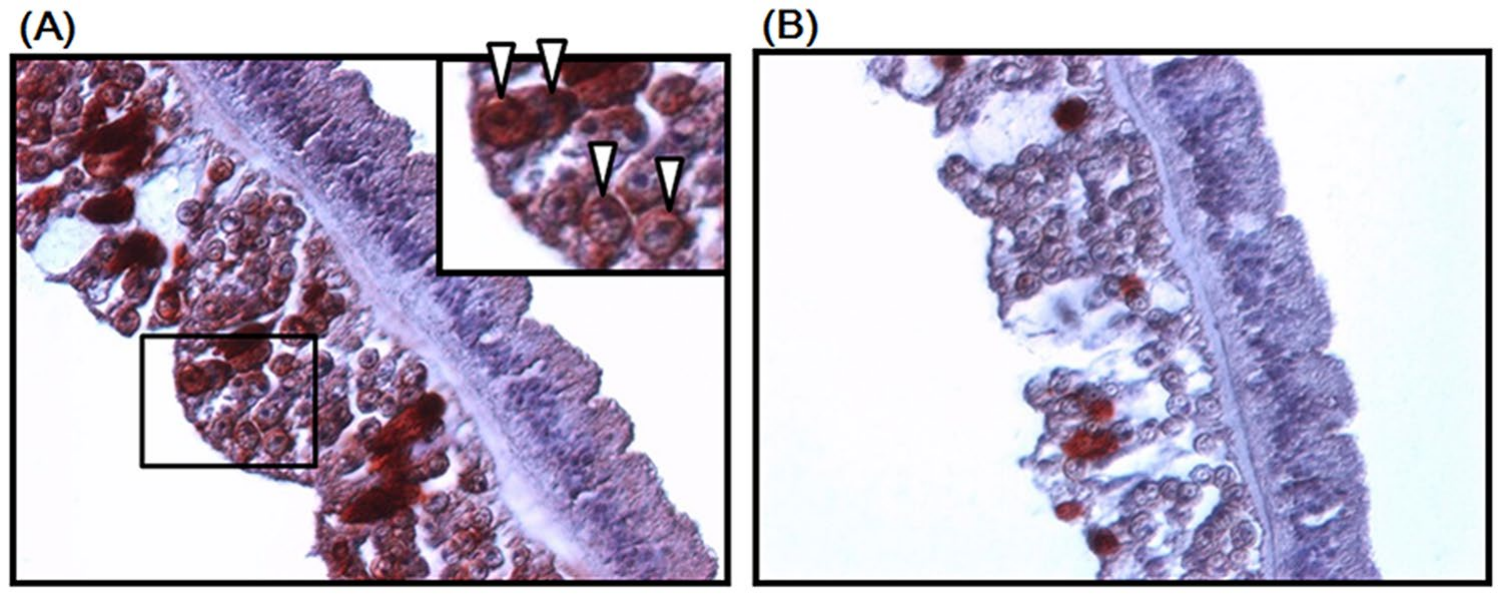
Immunohistochemical staining of tentacles of *E*. *glabrescens*. Paraffin-embedded sections of tentacle tissue were stained using (**A**) the 2–6F mAb and (**B**) pre-immune serum. The reaction was detected using 3-amino-9-ethylcarbazole chromogen stain (red), and the nuclei were counterstained with hematoxylin (blue) (magnification: 400×). Insert in (**A**) shows an enlargement of the boxed section. The arrowhead indicates positive staining in *Symbiodinium*.

Immunohistochemical examination was performed to identify the tissue or cellular targets of the 2–6F mAb. As shown in Fig. 2A, the symbionts in the gastroderm showed strong and positive signals, while no signals were observed in other cellular locations. These results agreed with those of the western blotting assay (Fig. 1B,C) and demonstrated that the 2–6F mAb specifically recognized symbiont proteins. Figure 2 also shows that the 2–6F mAb recognized *Symbiodinium* proteins not only on the cell surface but also in the cytoplasm.

**Figure 3:**
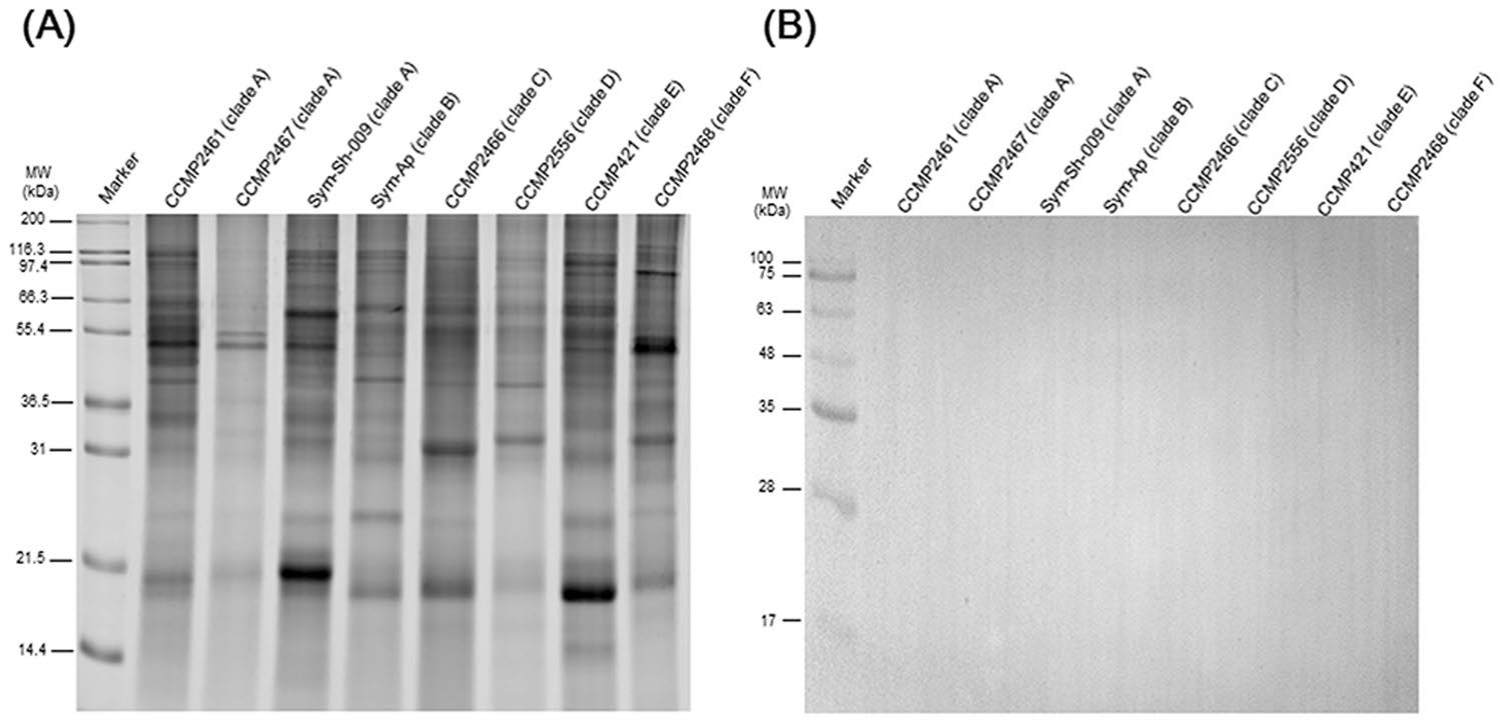
The 2–6F mAb did not recognize free-living *Symbiodinium* proteins. (**A**) Protein from eight types of free-living *Symbiodinium*(clade A,B,C,D,E,F) were analyzed by SDS-PAGE, and total proteins were stained with SYPRO^®^ Ruby. (**B**) Western blotting was performed using the 2–6F mAb.

**Figure 4:**
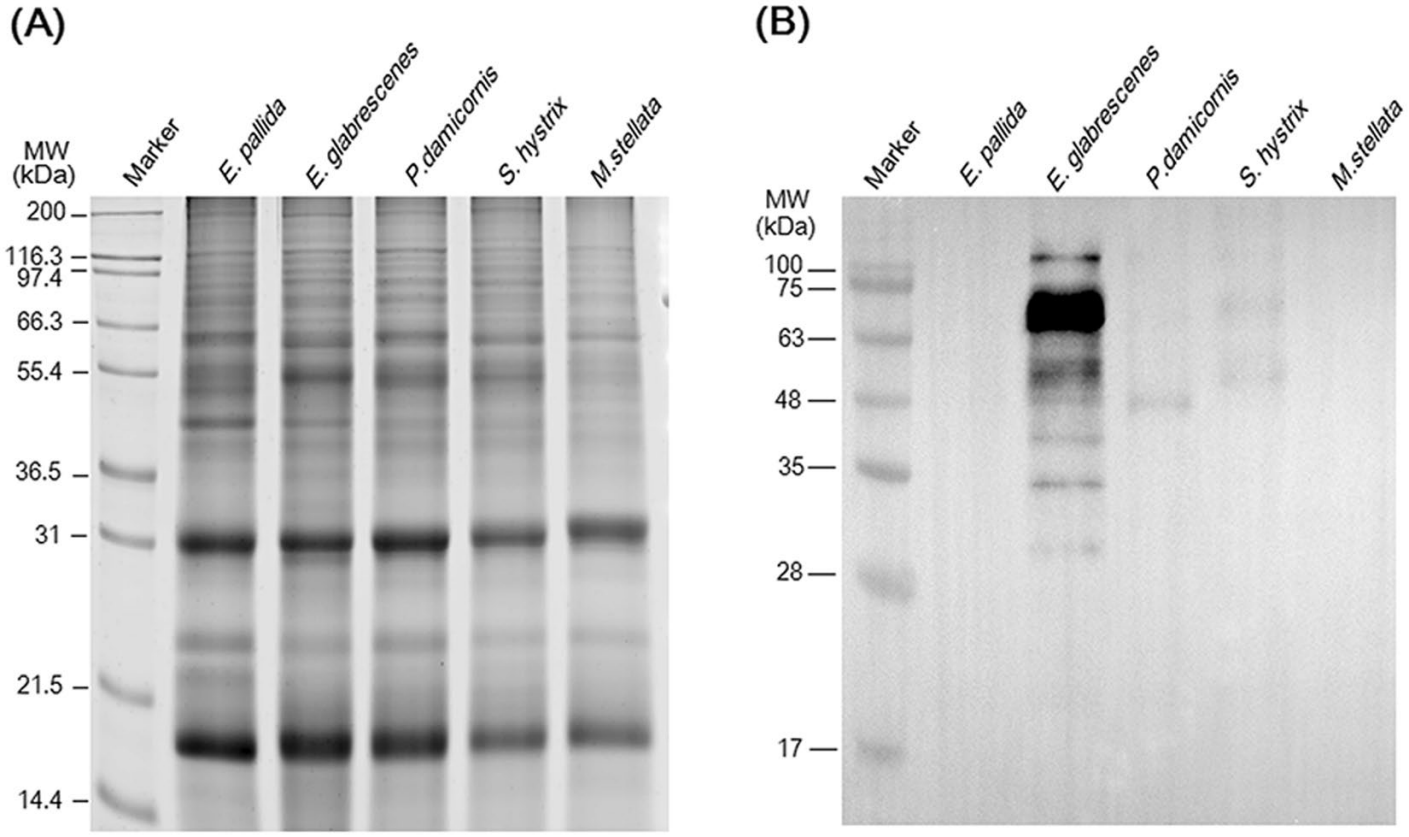
The 2–6F mAb only recognized clade C symbiotic *Symbiodinium* proteins and not those of clades (B and D). (**A**) Five types of symbiotic *Symbiodinium* protein were analyzed by SDS-PAGE, and total proteins were stained with SYPRO^®^ Ruby. (**B**) Western blotting was performed using the 2–6F mAb. *E*. *pallida*: total proteins of *Symbiodinium* isolated from *Exaiptasia pallida* (clade B); *E*. *glabrescens*: total proteins of *Symbiodinium* isolated from *E*. *glabrescens* (clade C); *P*. *damicornis*: total proteins of *Symbiodinium* isolated from *Pocillopora damicornis* (clade C); *S*. *hystrix*: total proteins of *Symbiodinium* isolated from *Seriatopora hystrix* (clade C); *M*. *stellata*: total proteins of *Symbiodinium* isolated from *Montipora stellata* (clade D).

To further examine the specificity of the 2–6F mAb toward *Symbiodinium per se*, different clades of free-living and symbiotic *Symbiodinium* proteins were further assessed by western blotting. The results showed that the 2–6F mAb did not react with cell lysates from free-living *Symbiodinium* of different clades (A, B, C, D, E, F; see Fig. 3 and Table 1). However, the 2–6F mAb only recognized lysates of clade C symbiotic *Symbiodinium* from *E*. *glabrescens*, *Pocillopora damicornis*, and *Seriatopora hystrix* (Fig. 4).

**Table 1:** Summary of the specificity of the 2–6F mAb by western blotting.

Clade	Free-living *Symbiodinium*		Symbiotic *Symbiodinium*	
	Strain Name	Result	Host Name	Result
A	CCMP2461	−		N/A
	CCMP2467	−		
	Sym-Sh-009	−		
B	Sym-AP	−	*Exaiptasia pallida*	−
C	CCMP2466	−	*Euphyllia glabrescens*	+
			*Seriatopora hystrix*	+
			*Pocillopora damicornis*	+
D	CCMP2556	−	*Montipora stellata*	−
E	CCMP421	−		N/A
F	CCMP2468	−		N/A

N/A = not analyzed. “+” = positive signals detected in western blotting. “−” = negative signals detected in western blotting.

### Epitopes of symbiont proteins recognized by the 2–6F mAb

**Figure 5:**
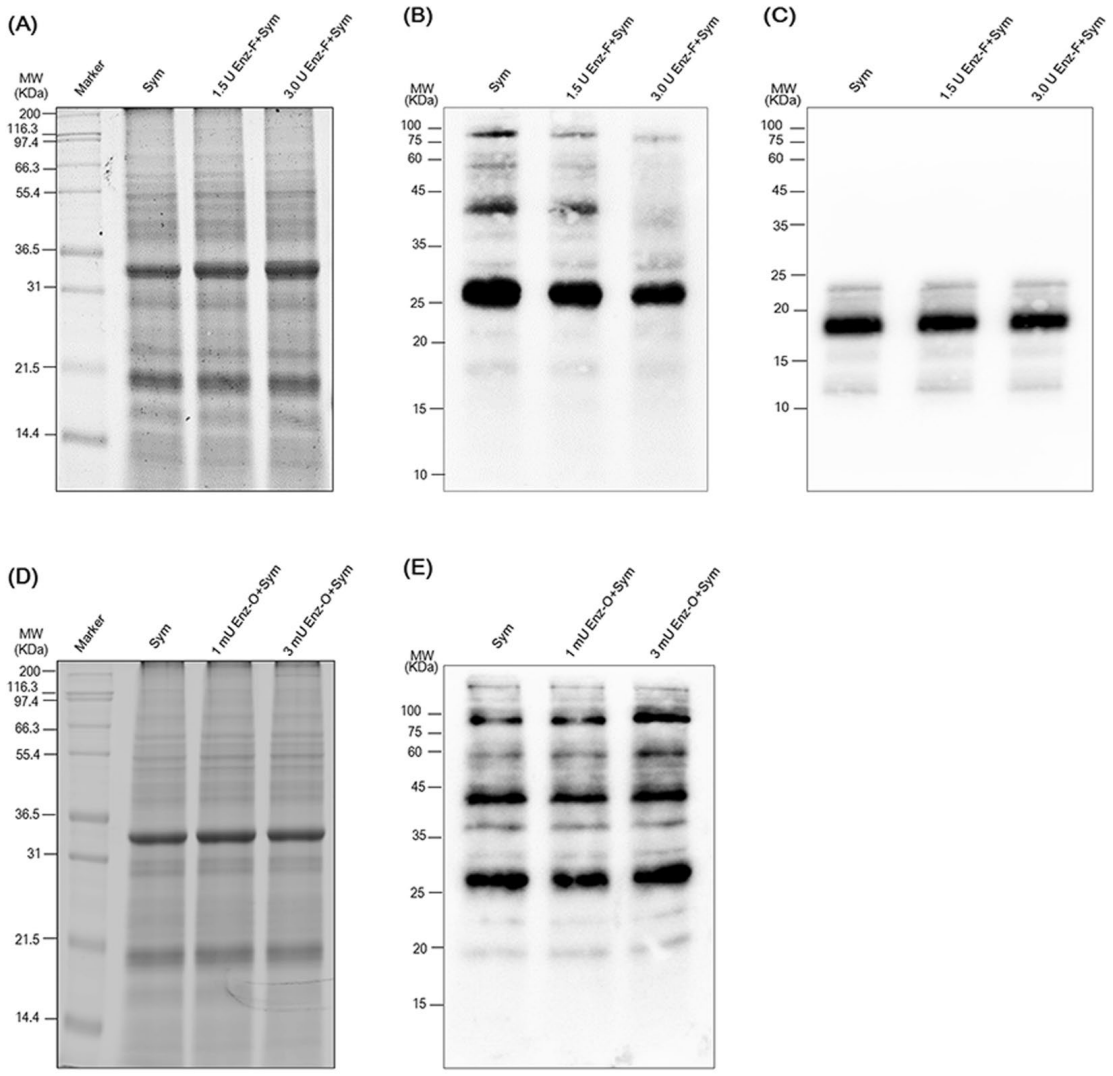
The 2–6F mAb recognized glycoproteins of symbiotic *Symbiodinium* proteins. (**A**) *Symbiodinium* proteins incubated with or without PNGase F were analyzed by SDS-PAGE and stained with SYPRO^®^ Ruby. The molecular weight of PNGase F is about 36 KDa. (**B**) and (**C**) Western blotting were performed using the 2–6F mAb and anti-light-harvesting protein antibodies. Sym: total proteins of *Symbiodinium* isolated from *E*. *glabrescens* (clade C); 1.5 U Enz-F + Sym: total proteins of *Symbiodinium* incubated with 1.5 U PNGase F at 37 °C for 1 h; 3.0 U Enz-F + Sym: total proteins of *Symbiodinium* incubated with 3.0U PNGase F at 37 °C for 1 h. (**D**) *Symbiodinium* proteins incubated with or without O-glycosidase were analyzed by SDS-PAGE and stained with SYPRO^®^ Ruby. The molecular weight of O-glycosidase is about 180 KDa. (**E**) Western blotting was performed using the 2–6F mAb. Sym: total proteins of *Symbiodinium* isolated from *E*. *glabrescens* (clade C); 1 mU Enz-O + Sym: total proteins of *Symbiodinium* incubated with 1 mU O-glycosidase at 37 °C overnight; 3 mU Enz-O + Sym: total proteins of *Symbiodinium* incubated with 3 mU O-glycosidase at 37 °C overnight.

The high selectivity and specificity of the 2–6F mAb toward proteins of symbiotic *Symbiodinium* indicate that epitopes of symbiont proteins are good candidates for symbiotic markers. Furthermore, as shown in Figs 1B and 4B, the 2–6F mAb recognized multiple bands of symbiont proteins, which is abnormal in comparison with a regular mAb that recognizes one single protein. Therefore, we speculated that the 2–6F mAb might recognize modified moieties of proteins, such as sugar, phosphate, ubiquitin, lipid, hydroxyl group, methyl group, and acetyl group^26, 27^. Glycoproteins on *Symbiodinium* cell walls have been shown to participate in the establishment of symbiotic relationships with sea anemones^28^, and glycosylation is one of the most common post-translational modifications of proteins in eukaryotic cells^29^. We predicted that the 2–6F mAb might recognize the glycan moiety of symbiont proteins. *O*-linked glycosylation occurs when glycans are attached to serine or threonine residues^30^, while *N*-linked glycosylation occurs when glycans are attached to asparagine^31^. To examine the linkage type of the glycoproteins, the peptide *N*-glycosidase F (PNGase F) was incubated with cell lysates, followed by 2–6F mAb western blotting as shown in Fig. 5. The results showed that PNGase F treatment abolished the antigenicity of symbiont protein toward the 2–6F mAb, which strongly indicated that the 2–6F mAb recognized *N*-linked glycans of symbiont proteins and not the peptide domains. The same experiment also performed by incubating *Symbiodinium* proteins with O-glycosidase, which remove the *O*-linked GalNAc chain from glycoprotein. The result showed that O-glycosidase had no effect on the antigenicity of *Symbiodinium* protein (Fig. 5D and E), which indicated that the epitopes of 2–6F is on the *N*-linked not *O*-linked glycans. Therefore, the epitopes of *Symbiodinium* proteins recognized by the 2–6F mAb were the *N*-linked glycan domains.

**Figure 6:**
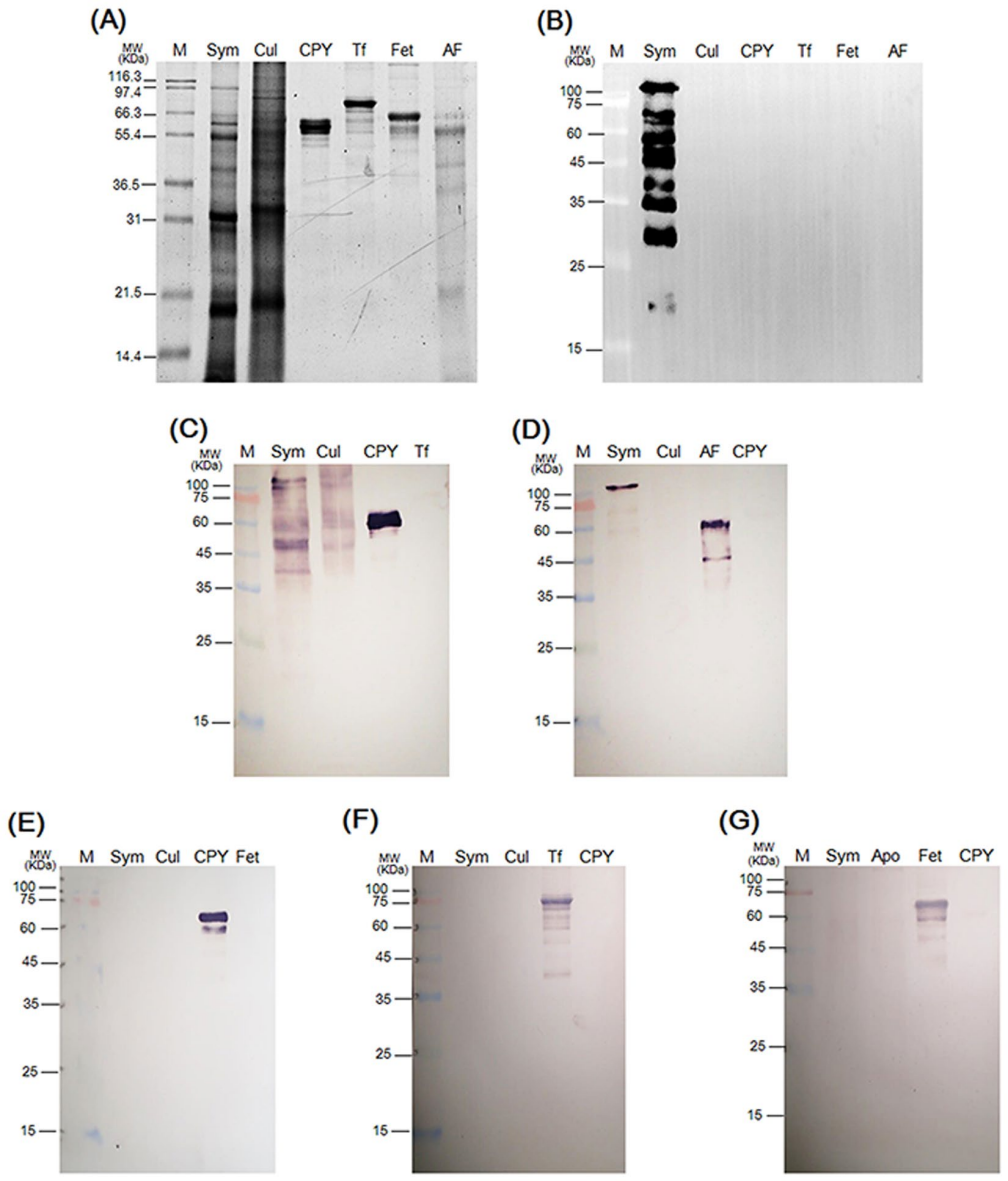
Detection of terminal sugar residues of glycoproteins in symbiotic and free-living *Symbiodinium*. (**A**) Six proteins (two *Symbiodinium* proteins and four control glycoproteins) were analyzed by SDS-PAGE and stained with SYPRO^®^ Ruby. (**B**) Western blotting was performed using the 2–6F mAb. Five types of lectin-conjugated alkaline phosphatase, (**C**) GNA, (**D**) PNA, (**E**) MAA, (**F**) SNA, and (**G**) DSA, were used to detect different glycans. M: marker; Sym: total proteins of symbiotic *Symbiodinium* (clade C) freshly isolated from *E*. *glabrescens*; Cul: total proteins of cultured *Symbiodinium* CCMP 2466 (clade C); CPY: carboxypeptidase Y; Tf: transferrin; Fet: fetuin; AF: asialofetuin. Four control glycoproteins were used: CPY, Tf, Fet, and AF. For GNA, CPY is a positive control, and Tf is a negative control. For PNA, AF is a positive control, and CPY is a negative control. For MAA, CPY is a positive control, and Fet is a negative control. For SNA, Tf is a positive control, and CPY is a negative control. For DSA, Fet is a positive control, and CPY is a negative control.

The nature of the terminal sugar residues of the recognized *N*-linked glycoproteins was then examined. A previous study showed that symbiosis-associated glycoproteins on *Symbiodinium* cell walls could bind to lectins, such as *Galanthus nivalis* agglutinin (GNA) and *Datura stramonium* agglutinin (DSA)^28^. To further explore which types of glycoproteins are recognized by the 2–6F mAb, five types of lectin [i.e., GNA, DSA, peanut agglutinin (PNA), *Maackia amurensis* agglutinin (MAA) and *Sambucus nigra* agglutinin (SNA)] were chosen to specifically identify the type of glycan moieties. As shown in Fig. 6C and D, symbiont proteins only bound to GNA and PNA, and the binding protein patterns obviously differed from those of the 2–6F mAb (Fig. 6B). Moreover, the 2–6F mAb did not bind to four control glycoproteins: carboxypeptidase Y, transferrin, fetuin, and asialofetuin (Fig. 6B). These results suggested that the 2–6F-recognized glycans differed from those recognized by the five lectins (GNA, PNA, MAA, SNA, and DSA). In other words, 2–6F did not recognize high mannose, galactose-β-(1–3)-N-acetyl-galactosamine linkages, galactose-α-(2–3)-sialic acid linkages, galactose-α-(2–6)-sialic acid linkages, or galactose-β-(1–4)-N-acetylglucosamine linkages. The types of glycans recognized by 2–6F remain to be elucidated.

### Glycoproteins in symbiotic and free-living *Symbiodinium*

Logan and colleagues found that concanavalin A significantly bound to cell surface glycans of eight *Symbiodinium* cultures^32^, which suggested a widespread distribution of mannose residues in *Symbiodinium*, in agreement with the present result. However, in the previous study, only clades A, B, D, E, and F *Symbiodinium* were examined; clade C *Symbiodinium* was not analyzed. In addition, Wood-Charlson and coworkers provided evidence that the surface of symbiotic clade C1f *Symbiodinium* contains α-mannose/α-glucose and α-galactose^33^. That evidence and our results both indicated that the major type of glycan in free-living clade C *Symbiodinium* glycoproteins is mannose. As shown in Fig. 6C and D, the symbiont proteins showed positive signals toward GNA (high mannose or hybrid-type carbohydrate chains) and PNA (galactose-β-(1–3)-N-acetyl-galactosamine linkage). However, the free-living *Symbiodinium* proteins only reacted with GNA, indicating a mannose nature of glycans. The binding patterns in symbiont proteins were obviously altered, strongly suggesting that the glycan moiety of symbiont glycoproteins is altered. These results demonstrated that the glycan moiety of *Symbiodinium* is changed during the process of cnidaria-*Symbiodinium* endosymbiosis, which may be induced by host regulation during post-translational modification.

Most studies of the effects of protein modification in coral/dinoflagellate symbiosis have focused on lectin/glycan recognition^28, 32, 33^, and no study has discussed the differences in glycoproteins between symbiotic and free-living *Symbiodinium*. The present study examined the different glycan types in symbiotic and free-living *Symbiodinium* and provided a new aspect and a new tool by which to study coral/dinoflagellate symbiosis.

### Symbiotic markers

**Figure 7:**
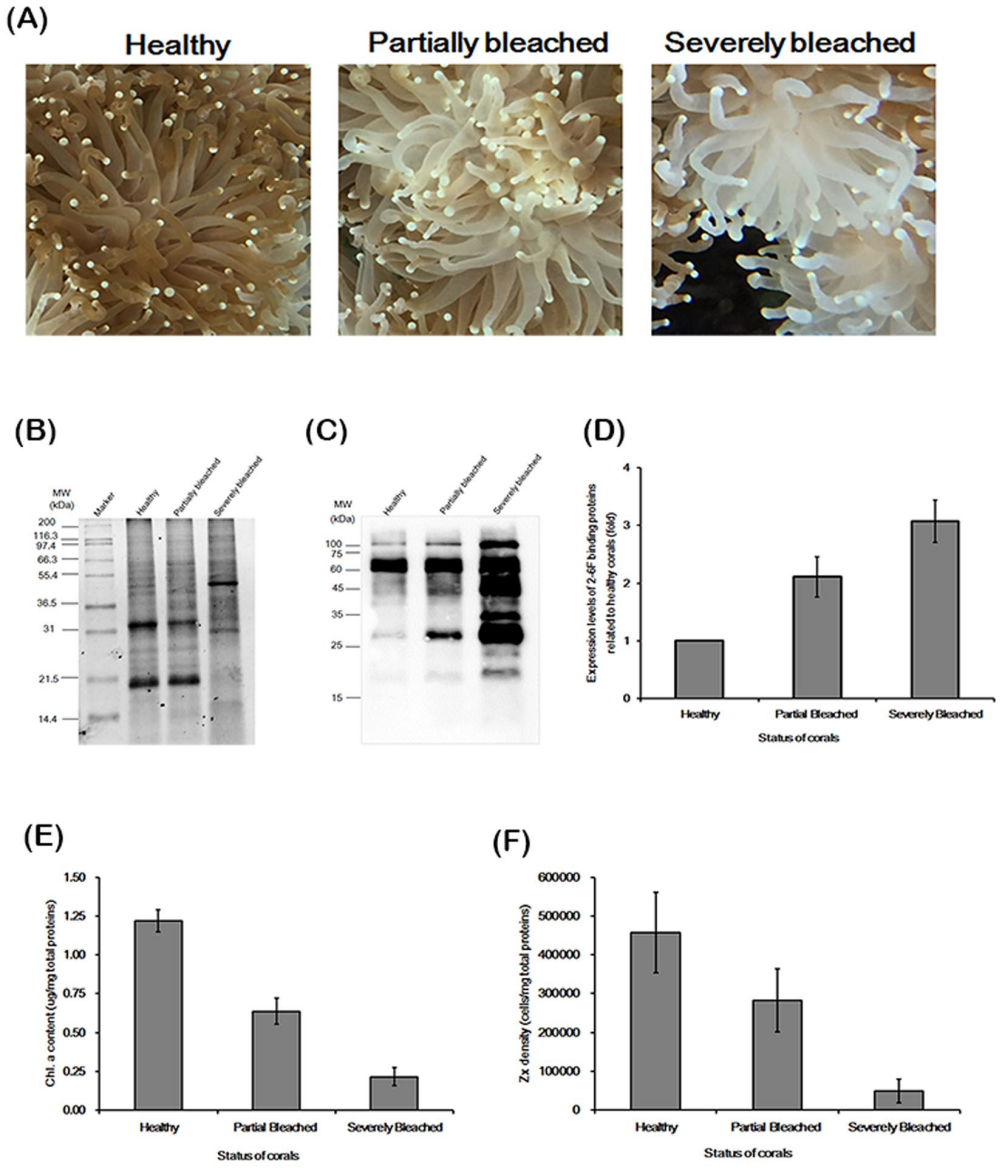
The expression levels of 2–6F binding proteins correlated with their symbiotic states.(**A**) The appearances of healthy, partially bleached, and severely bleached *E*. *glabrescens*. (**B**) Total proteins of *Symbiodinium* isolated from healthy, partially bleached, and severely bleached *E*. *glabrescens* were analyzed by SDS-PAGE and stained with SYPRO^®^ Ruby. (**C**) Western blotting was performed using the 2–6F mAb. Healthy: total proteins of *Symbiodinium* isolated from healthy *E*. *glabrescens*; Partially Bleached: total proteins of *Symbiodinium* isolated from partially bleached *E*. *glabrescens*; Severely Bleached: total proteins of *Symbiodinium* isolated from severely bleached *E*. *glabrescens*. These data are representative of three independent experiments. (**D**) Quantification of the western blotting results of (**C**) by Image J software; N = 3, P < 0.05 between three different coral healthy states. (**E**) Zooxanthellate density of total proteins from different coral healthy states; N = 3, P < 0.05 between three different coral healthy states. (**F**) Chlorophyll *a* content of total proteins from corals of different health states; N = 3, P < 0.001 between three different coral healthy states. Healthy: healthy *E*. *glabrescens*; Partially Bleached: partially bleached *E*. *glabrescens*; Severely Bleached: severely bleached *E*. *glabrescens*.

To further demonstrate the feasibility of using the 2–6F mAb to identify symbiotic marker proteins, corals of differing symbiosis status (i.e., severely bleached, partially bleached, and normal) were evaluated by the 2–6F mAb, as shown in Fig. 7A. The expression levels of 2–6F binding proteins were correlated with the symbiotic relationship in a dose-dependent manner. Severely bleached corals had the highest expression levels of 2–6F-binding proteins, and healthy coral had the lowest expression levels (Fig. 7B–D). It is possible that, in order to counteract bleaching, coral needs to express more symbiotic-related proteins, which leads to a higher 2–6F mAb signal. The 2–6F binding proteins are glycoproteins and exist both on the cell surface and in the cytoplasm of *Symbiodinium* (Fig. 2). Glycoproteins are often important integral membrane proteins, where they play a role in cell–cell interactions. A previous study showed that glycoproteins play an important role in recognition in coral/dinoflagellate endosymbiosis^28, 32, 33^. In severely bleached corals, 2–6F-binding proteins may be expressed at high levels to strengthen the cell–cell interactions between the host and *Symbiodinium* cells to prevent more *Symbiodinium* cells from leaving the host cells. However, glycoproteins in the cytosol and nucleus can be modified through the reversible addition of a single GlcNAc residue to regulate cell signaling inside the cells^34^. In severely bleached corals, host corals experience crisis, and they need to transmit more cell signals and express more proteins (such as heat-shock proteins, apoptosis-related proteins, and cell cycle proteins) to manage the crisis. Thus, 2–6F binding proteins in the cytosol may be responsible for regulating cell signaling and are expressed at high levels in severely bleached corals.

Many genes and proteins that have higher expression levels in bleached corals than in healthy corals have been reported. In terms of gene levels, 191 key genes were found to be up-regulated in bleached corals, and their functions involve cell signaling, oxidative stress, calcium homeostasis, cytoskeletal organization, cell death, calcification, metabolism, protein synthesis, heat shock protein activity, and transposon activity^35^. In terms of proteins levels, 13 key proteins were found to have significantly higher protein expression in bleached corals than in unbleached corals, and their functions involve stress response, UV response, apoptosis, cytoskeleton, and cell cycle proteins^36^. The 2–6F binding proteins might have similar functions to the above-described proteins, and the detailed functions of 2–6F binding proteins require further investigation.

The western blotting analysis results shown in Fig. 1B indicated that at least seven major proteins are specific to symbiotic clade C *Symbiodinium*. As shown in Figs 4B, 5B, 6B and 7C, the molecular weights of these seven proteins were the same, but the intensity patterns of the protein bands were different. We surmised that the different intensity patterns of the protein bands was caused by different levels of post-translational modification. The *Symbiodinium* proteins shown in the different figures were freshly isolated from different colonies of *E*. *glabrescens* at different times. According to the results shown in Fig. 7, the different intensity patterns of protein bands may also be caused by different symbiotic states. Changes in expression levels of 2–6F binding proteins could be directly regulated by the symbiotic status. Corals used in the present study were maintained in the same aquarium tank. Consequently, environmental factors that might affect the glycoprotein level^37^, such as temperature, light and salinity, could be eliminated.

### Future applications of the 2–6F mAb in endosymbiosis studies

Few studies have investigated cnidarian-*Symbiodinium* symbiosis using custom antibodies. In 2001, Wakefield and Kempf^38^ first produced anti-host and anti-symbiont monoclonal antibodies and demonstrated that the outer layer of the symbiosome membrane is host-derived and the inner layer is symbiont-derived. Then, Schwarz and Weis^39^ produced an anti-symbiosis-related protein (sym32) antibody of the sea anemone *Anthopleura elegantissima* and demonstrated that when *Symbiodinium* is engulfed into host cells, the host sym32 protein relocates from gastrodermal vesicles to the symbiosome membrane. In 2005, Puverel *et al*.^40^ produced an anti-soluble organic matrix polyclonal antibody of *Stylophora pistillata* to study skeletogenesis and coral biomineralization in scleractinian corals. The above-described custom antibodies recognized both the symbiotic and aposymbiotic states of the cnidarian; therefore, these antibodies cannot be symbiotic markers. However, the 2–6F mAb specifically recognized clade C symbiotic *Symbiodinium* and could therefore be a symbiotic indicator. In the future, we plan to infect bleached *Aiptasia pulchella* (now re-named *Exaiptasia pallida*)^41^ and observe the change in *Symbiodinium* from the aposymbiotic to the symbiotic state, using the 2–6F mAb to study the location and initiation of cnidarian-dinoflagellate endosymbiosis. The use of the 2–6F mAb will increase our knowledge of the mechanism of cnidaria-dinoflagellate endosymbiosis.

## Methods

### Reagents and culture media

Iscove’s modified Dulbecco’s medium (IMDM, pH 7.4) (Thermo Fisher Scientific, Waltham, MA, USA), filtered seawater (FSW), and artificial seawater (ASW) were prepared follow the processes described in a previous study^18^.

### Coral collection and maintenance

*E*. *glabrescens* colonies were collected by SCUBA divers and maintained in the husbandry center of the National Museum of Marine Biology and Aquarium (NMMBA). Detailed information regarding the collection and maintenance was presented in a previous study^18^. *Pocillopora damicornis*, *Seriatopora hystrix*, *Montipora stellate*, and *Exaiptasia pallida* have been cultured in the husbandry center of the NMMBA for many years.

### Isolation of SGCs

SGCs were isolated according to the method described in a previous study^18^, and the intactness of the SGC plasma membranes was examined as previously described^42^.

### Monoclonal antibody production, screening, and immunoglobulin isotype identification

BALB/cJ mice were immunized intraperitoneally every two weeks (a total of 4 times) with isolated SGCs (5 × 10^7^ SGCs/mouse) emulsified with an equal volume of Freund’s complete/incomplete adjuvant. Three days before fusion, mice were boosted intravenously with SGCs in PBS (5 × 10^7^ SGCs/100 µL/mouse). Splenocytes (1 × 10^8^ cells) from the immunized mice were fused with FO myeloma cells (2 × 10^7^ cells) in the presence of PEG 1500 (Sigma-Aldrich, St. Louis, MO, USA). After fusion, the cells were re-suspended in complete medium containing hypoxanthine/aminopterin/thymidine (HAT), and the cells were then distributed into 96-well plates and cultured for 14 days. Positive clones were selected by the enzyme-linked immunosorbent assay (ELISA) method with total proteins of SGCs, and single clones were obtained using a limiting dilution assay. The SGC proteins were defined as total proteins of SGCs, which were isolated from *E*. *glabrescens* and extracted. Hybridoma cells were maintained in Dulbecco’s Modified Eagle’s medium (Thermo Fisher Scientific) containing 15% FBS, 1% penicillin/streptomycin, 2% L-glutamine, and 1% adjusted NaHCO_3_ solution. The isotype of 2–6F was IgG_1_, and the light chain was a kappa chain, as determined using a mouse isotyping kit (cat. no LFM-ISO-1-5, RayBiotech, Norcross, GA, USA). The 2–6F antibody was purified from the culture medium using protein A chromatography (GE Healthcare, Piscataway, NJ, USA).

### Culture of free-living *Symbiodinium*

All free-living *Symbiodinium* samples used in this study were purchased from the Provasoli-Guillard National Center for Culture of Marine Phytoplankton (Bigelow Laboratory for Ocean Science, East Boothbay, ME, USA) and included clade A (CCMP2461, CCMP2467, Sym-Sh-009), clade B (Sym-AP), clade C (CCMP2466), clade D (CCMP2556), clade E (CCMP421), and clade F (CCMP2468). The cells were cultured in F/2 medium (Sigma-Aldrich) containing antibiotics (100 μg/ml streptomycin and 100 U/ml penicillin) at 25 °C under a 12 h light, 12 h dark regime.

### Isolation of epidermal cells, gastrodermal cells, and *Symbiodinium* from *E*. *glabrescens*

Fifty tentacles from *E*. *glabrescens* were collected as described above. After rinsing with FSW, tentacle tips were removed using micro scissors (Spring Type, AESCULAP, Center Valley, PA, USA) to decrease the presence of nematocytes and prevent their interference during the isolation process. The epidermis and gastrodermis were then separated using ASW containing 3% N-acetylcysteine (pH 8.2) as previously described^43^. Epidermal cells were collected for western blotting. Gastrodermal cells were incubated in 2 mL of ASW containing 1× complete protease inhibitor cocktail (25× stock solution in 2 mL ddH_2_O, Cat. 11697498001; Roche, Madison, WI, USA) and homogenized on ice with ten passes of a 7-mL glass tissue grinder (Kimble Chase, Vineland, NJ, USA). The crude homogenate was then added in a discontinuous Percoll (GE Healthcare) gradient step (100%, 80%, 60%, 40%, and 20%, 500 × *g* for 20 min). The *Symbiodinium* cells were collected from the 80–100% interface of the discontinuous Percoll gradient centrifugation step, and the host cells were collected from the 20–40% range. The collected cells were washed with ASW for western blotting.

### Protein extraction of different cell lysates

Total proteins of SGCs, *Symbiodinium*, epidermal cells, and gastrodermal cells were extracted as follows. The different cells were broken by glass beads (Sigma cat. no G9268) using a mixer mill. The supernatant was collected (215,000 × *g* for 10 min at 4 °C) and precipitated by 20% trichloroacetic acid overnight at −20 °C. Precipitated proteins were then collected (215,000 × *g* for 15 min at 4 °C) and washed sequentially with ice-cold 1% DTT/acetone twice and acetone (10 min at 4 °C for each wash). The pellet was vacuum-dried (5,000 × *g*) at room temperature for 5–10 min, re-suspended in 1× SDS-PAGE sample buffer (62.5 mM Tris-HCl [pH 6.8], 2% SDS, 10% glycerol, and 10 mM DTT), and then quantified using a Qubit® Protein Assay Kit and Qubit® 2.0 fluorometer (Thermo Fisher Scientific) according to the manufacturer’s recommendations.

### Western blotting

Twenty micrograms of each protein sample were subjected to 12% SDS-PAGE and then blotted onto PVDF membranes (Immobilon-PSQ 0.45 mm; Millipore, Germany). The membranes were incubated in 5% skim milk / PBST buffer (0.1% Tween-20, 137 mM NaCl, 2.7 mM KCl, 10 mM Na_2_HPO_4_, 2 mM KH_2_PO_4_) at RT for 1 h, followed by incubation with rabbit anti-ribulose-1, 5-bisphosphate carboxylase/oxygenase (rubisco) large subunit (1:2,000 dilution; cat. no AS03 037, Agrisera, Vannas, Sweden; a marker for the presence of *Symbiodinium* protein), mouse anti-actin (1:10,000 dilution; cat. no MAB1501, Millipore; a marker for the presence of host coral), mouse anti-light harvest protein (1:10,000 dilution; custom antibody, an internal control for the *Symbiodinium* proteins) and pre-immune serum (1:5,000 dilution) or mouse anti-2–6F (1:5,000 dilution) antibodies in PBST buffer at 4 °C overnight. The membranes were then washed five times with PBST buffer for 10 min each time and incubated with HRP-conjugated goat anti-rabbit or anti-mouse IgG antibodies (Millipore) in PBST buffer (1:5,000 dilution for each secondary antibody). The membranes were subsequently washed with PBST buffer and visualized using a SuperSignal West Pico Chemiluminescent substrate kit (cat. no 34080, Thermo Fisher Scientific) according to the manufacturer’s recommendations.

### Immunohistochemical stain (IHC stain)

Amputated *E*. *glabrescens* tentacles were fixed in 4% paraformaldehyde overnight at 4 °C and then washed three times with PBS at 4 °C. The fixed tissues were washed with water and dehydrated with increasing concentrations of ethanol (50, 70, 80, 90, 95, and 100%). The solvent was changed to paraffin in a graded xylene/paraffin series (100%, 50%, 0%; 20 min each step). The tissues were embedded in a paraffin block and cut into 5-μm-thick sections. The paraffin on the tissues was removed using xylene, and the tissues were rehydrated using ethanol. The slides were blocked with background-reducing components (DakoCytomation, Carpinteria, CA, USA) for 10 min and then incubated with purified 2–6F (1:200 dilution) antibody at 4 °C overnight. The slides were then treated consecutively using an LSAB2 system-HRP kit (DakoCytomation, cat. no K0672). After incubation using 3-amino-9-ethylcarbazole chromogen (AEC) substrate chromogen (DakoCytomation), the slides were washed and counterstained with Mayer’s hematoxylin (Biocare Medical, Concord, CA, USA). For negative controls, pre-immune serum (1:200 dilution) was used as the primary antibody to demonstrate the background staining.

### Detection of glycoproteins

Proteins of symbiotic and free-living *Symbiodinium* were subjected to 12% SDS-PAGE and then blotted onto PVDF membranes. The detection of glycoproteins on the PVDF membrane was performed using a DIG glycan differentiation kit (Roche, cat. no 11-210-238-001, Mannheim, Germany). In brief, the PVDF membrane was blocked in blocking buffer for at least 30 min and then incubated with different lectin solutions [*Galanthus nivalis* agglutinin (GNA), peanut agglutinin (PNA), *Maackia amurensis* agglutinin (MAA), *Sambucus nigra* agglutinin (SNA), or *Datura stramonium* agglutinin (DSA)] for 1 h. After washing with TBS buffer three times, the membrane was incubated with anti-digoxigenin-alkaline phosphatase solution for 1 h and stained with a substrate solution (4-nitro blue tetrazolium chloride and 5-bromo-4-chloro-3-indolylphosphate). In this method, different agglutinins specifically bind to different types of sugar linkages. For example, GNA can bind to high mannose or hybrid-type carbohydrate chains; PNA can bind to a galactose-β-(1–3)-*N*-acetyl-galactosamine linkage; MAA can bind to a galactose-α-(2–3)-sialic acid linkage; SNA can bind to a galactose-α-(2–6)-sialic acid linkage; and DSA can bind to a galactose-β-(1–4)-*N*-acetylglucosamine linkage. Four control glycoproteins were used: carboxypeptidase Y (for binding with GNA), transferrin (for binding with SNA), fetuin (for binding with MAA and DSA), and asialofetuin (for binding with PNA). Carboxypeptidase Y, a vacuolar enzyme from *Saccharomyces cerevisiae*, has four oligosaccharide chains linked to asparagine in the glycoprotein. The mannosidase-resistant phosphorylated oligosaccharide has the structure Man → P → 6αMan → αMan → 6βMan → 4GlcNAcH_2_, in which some of the phosphate groups are substituted with mannobiose instead of mannose^44^. Transferrins are glycoproteins that can reversibly bind two iron atoms. They are responsible for iron transfer to reticulocytes for the synthesis of hemoglobin^45^. The structure of transferrins contains two identical, branched heterosaccharide chains (glycans), and an examination of these two *N*-linked glycosylation sites has demonstrated that both glycans are bound through a 4-*N*-(2-acetamido-2-deoxy-β-D-glucopyranosyl)-L-asparagine linkage^46^. However, few transferrins with solely tri-branched glycans exist^47, 48^. Fetuin is the major glycoprotein in fetal calf serum. Spiro and co-workers^49, 50^ showed that fetuin has three oligosaccharide chains per molecule *O*-glycosidically linked to serine or threonine. However, 13 years later, Spiro and co-workers examined the *O*-linked carbohydrate units by gel filtration, thin-layer and anion-exchange chromatography and identified the presence of an *O*-glycosidically linked hexasaccharide in fetuin. The structure of the hexasaccharide was determined to be NeuAcα2 → 3Galβ1 → 3[NeuAcα2 → 3Galβl → 4GlcNAcβ1 → 6]GalNAc^51^. Asialofetuin is a glycoprotein that has three asparagine-linked triantennary complex carbohydrate chains with terminal LacNAc residues^52^ and has the *O*-linked carbohydrate structure of Galβ1 → 3GalNAcα1 → Ser (Thr)-protein^53^.

### Statistical analysis

Experimental data were examined using one-way ANOVA. P < 0.05 was considered statistically significant.

## Conclusion

A new monoclonal antibody, 2–6F, was generated that specifically recognizes clade C symbionts but not their free-living counterpart or other *Symbiodinium* clades. The epitopes of 2–6F were glycans of *N*-linked glycoproteins on symbionts, and the glycan types are not high mannose, galactose-β-(1–3)-*N*-acetyl-galactosamine linkage, galactose-α-(2–3)-sialic acid linkage, galactose-α-(2–6)-sialic acid linkage, or galactose-β-(1–4)-*N*-acetylglucosamine linkage. The precise type of glycan recognized by the 2–6F mAb remains to be elucidated. The 2–6F mAb-recognized proteins are symbiotic markers for clade C symbionts whose expression levels vary depending on the health status of corals. Furthermore, application of the 2–6F mAb in this study demonstrated that glycoproteins between symbiotic and free-living *Symbiodinium* are distinctly different. The glycan profiles of the symbiont populations were distinct from those of the cultured *Symbiodinium*, suggesting a host regulation mechanism by post-translational modification during the endosymbiotic interaction.
